# Construction of circRNA-mediated ceRNA network and immunoassay for investigating pathogenesis of COPD

**DOI:** 10.3389/fgene.2024.1402856

**Published:** 2024-09-03

**Authors:** Ting Yang, Wenya Xu, Jie Zhao, Jie Chen, Siguang Li, Lingsang Lin, Yi Zhong, Zehua Yang, Tian Xie, Yipeng Ding

**Affiliations:** ^1^ Department of General Practice, Hainan Affiliated Hospital of Hainan Medical University, Hainan General Hospital, Haikou, Hainan, China; ^2^ Zayun Township Health Center, Qiongzhong Li and Miao Autonomous County, Haikou, Hainan, China; ^3^ Department of Pulmonary and Critical Care Medicine, Hainan General Hospital, Hainan Affiliated Hospital of Hainan Medical University, Haikou, Hainan, China

**Keywords:** chronic obstructive pulmonary disease, circDTL, hsa-miR-330-3p, CCL20, competitive endogenous RNA

## Abstract

**Background:**

The chronic respiratory condition known as chronic obstructive pulmonary disease (COPD) was one of the main causes of death and disability worldwide. This study aimed to explore and elucidate new targets and molecular mechanisms of COPD by constructing competitive endogenous RNA (ceRNA) networks.

**Methods:**

GSE38974 and GSE106986 were used to select DEGs in COPD samples and normal samples. Cytoscape software was used to construct and present protein-protein interaction (PPI) network, mRNA-miRNA co-expression network and ceRNA network. The CIBERSORT algorithm and the Lasso model were used to screen the immune infiltrating cells and hub genes associated with COPD, and the correlation between them was analyzed. COPD cell models were constructed *in vitro* and the expression level of ceRNA network factors mediated by hub gene was detected by reverse transcription-quantitative polymerase chain reaction (RT-qPCR).

**Results:**

In this study, 852 differentially expressed genes were screened in the GSE38974 dataset, including 439 upregulated genes and 413 downregulated genes. Gene clustering analysis of PPI network results was performed using the Minimum Common Tumor Data Element (MCODE) in Cytoscape, and seven hub genes were screened using five algorithms in cytoHubba. CCL20 was verified as an important hub gene based on mRNA-miRNA co-expression network, GSE106986 database validation and the analysis of ROC curve results. Finally, we successfully constructed the circDTL-hsa-miR-330-3p-CCL20 network by Cytoscape. Immune infiltration analysis suggested that CCL20 can co-regulate immune cell migration and infiltration through chemokines CCL7 and CXCL3. *In vitro* experiments, the expression of circDTL and CCL20 was increased, while the expression of hsa-miR-330-3p was decreased in the COPD cell model.

**Conclusion:**

By constructing the circDTL-hsa-miR-330-3p-CCL20 network, this study contributes to a better understanding of the molecular mechanism of COPD development, which also provides important clues for the development of new therapeutic strategies and drug targets.

## 1 Introduction

Chronic Obstructive Pulmonary Disease (COPD) is a chronic respiratory disease characterized by persistent respiratory symptoms and limited airflow (Vestbo et al., 2013), with no obvious symptoms in the early stages. Most patients are diagnosed and treated when the disease has already progressed to the middle and late stages, often resulting in missed opportunities for optimal treatment, leading to a poor prognosis and increased mortality rate (Raherison and Girodet, 2009). The annual death toll from COPD is nearly three million, leading to a significant healthcare burden (Barnes et al., 2003). However, the pathogenesis of COPD remains poorly understood (Agustí et al., 2020), and there is an urgent need to investigate and clarify new targets and molecular mechanisms of COPD.

The ceRNA network hypothesis suggests that competing endogenous RNA (ceRNA) can competitively bind microRNAs, thereby indirectly affecting the expression of targeted genes (Long et al., 2019). circRNA is a noncoding RNA that can bind to miRNA and exert negative regulatory effects on their activity (Ebbesen et al., 2017). Increasing evidence suggests that circRNA-mediated ceRNA network plays a crucial role in various respiratory diseases (Lv et al., 2018). For instance, hsa_circ_0000003 is found to promote the progression of non-small cell lung cancer (NSCLC) by regulating the miR-338-3p/insulin receptor substrate 2 (IRS2) axis (Li et al., 2019). However, the study of the relationship between the circRNA-related ceRNA network and the development of COPD is still in its early stages. Recent studies have shown that immune infiltrating cells are strongly associated with the onset and progression of lung diseases (Zhong et al., 2021). For example, AL035458.2/hsa-miR-181a-5p/NCAPG2 can partially impact the prognosis of lung adenocarcinoma (LUAD) patients through immune infiltration (Chen et al., 2022). However, current studies have not focused on the regulatory mechanisms between infiltrating immune cells in COPD and ceRNA networks.

In this study, we identified DEGs in samples of COPD and normal samples on the basis of the GEO database. Then, we constructed the PPI network using the STRING database and utilized Cytoscape software to find COPD-related cluster modules. The cytoHubba plug-in within Cytoscape was utilized to identify hub genes using five algorithms (degree, MCC, MNC, DMNC, and clustering coefficient). Afterward, ceRNA networks were constructed using the starBase database and Cytoscape software. Additionally, immune infiltration analyses related to COPD were performed. Finally, the expression of key genes in the ceRNA network was detected in a COPD cell model. Our findings may provide a novel theoretical framework for elucidating the pathogenesis of COPD and offering innovative perspectives for biomarker screening in this disease.

## 2 Materials and methods

### 2.1 Data collection

In the NCBI’s Gene Expression Omnibus (GEO; http://www.ncbi.nlm.nih.gov/geo/), we first selected the miRNA dataset (GSE38974) for differential miRNA screening, used online database to predict genes, and used mRNA dataset (GSE106986) to validate genes. The dataset GSE38974 comprised of 23 samples from samples of COPD and 9 samples from healthy controls, whereas GSE106986 comprised of 14 COPD samples and 5 normal samples.

### 2.2 Identification of differentially expressed genes (DEGs)

To elucidate the differentially expressed genes (DEGs) that are associated with the onset and progression of COPD, differential analysis was performed using the Limma package in R software (Ritchie et al., 2015). The pheatmap package was used for visualization of heatmaps, while the ggplot2 package was used for volcano plots. As for the screening criteria for DEGs, we used |logFC| > 1 and *p* < 0.05 as the screening criteria. This criterion is commonly used in gene expression analysis because it can identify genes with significant and statistically significant changes in expression. And we used the Benjamini–Hochberg method to correct for *p*-values to control for false discovery rate.

### 2.3 Enrichment analysis

Gene Ontology (GO) provides a comprehensive understanding of gene function in three ways: cellular component (CC), molecular function (MF), and biological process (BP) (Blake et al., 2015). The Kyoto Encyclopedia of Genes and Genomes (KEGG) is utilized to identify significant enrichment in gene collections associated with specific KEGG pathways, providing information on a wide range of metabolic pathways, signaling pathways, and disease-related pathways (Kanehisa and Goto, 2000; Chen et al., 2017). GSEA enables the assessment of gene distribution patterns within a predefined gene set within a ranked gene table, based on their degree of association with the phenotype. This allows for the evaluation of their contribution to the phenotype (Subramanian et al., 2005). In this study, the clusterProfiler package in R software was utilized for conducting GO, KEGG, and GSEA analyses on the DEGs. The enrichment results were then visualized and significantly enriched functions and pathways were selected with a *p*-value < 0.05.

### 2.4 Construction of the PPI network

All DEGs were uploaded to the STRING online website (https://string-db.org/) (Szklarczyk et al., 2019), and PPI networks were constructed using a filter condition (combined score ≥ 0.7). Then, the eligible genes from the interaction file were imported into Cytoscape (v3.8.0) software for visualization (Shannon et al., 2003). PPI network results were analyzed for gene clustering using the Minimal Common Oncology Data Elements (MCODE) in Cytoscape to find important genes and modules (Parameters were set as Network Scoring: Degree Cutoff = 2, Cluster Finding: Node Score Cutoff = 0.2, K-Core = 2, Max. Depth = 100) (Zhou et al., 2021). Finally, we used five algorithms (Degree, MCC, MNC, DMNC, and Clustering Coefficient) in cytoHubba to screen key networks and selected the top 70 genes from each algorithm. Finally, we obtained the final hub gene by taking the intersection of these genes.

### 2.5 Construction mRNA-miRNA co-expression network

We performed miRNA differential expression analysis in COPD using the GSE24709 microarray dataset from the GEO database, identified target miRNAs of hub genes through the StarBase (version 3.0) database and selected miRNAs by intersecting the search results with differentially expressed genes. The mRNA-miRNA co-expression network was constructed using Cytoscape and visualized to illustrate the correlations between miRNAs and mRNAs.

### 2.6 GEO database validation and ROC curves

The GSE106986 dataset (including 5 COPD samples and 14 normal samples) was select chosen to validate the expression levels of hub genes in the COPD and normal group. We utilized the R programming language, specifically the ggplot2 and ggsignif packages, to generate boxplots and conducted statistical analysis using Student's t-test. Then, the ROC package in R software was employed to generate ROC curves by utilizing the expression levels of the identified hub genes. The area under the curve (AUC) represents a combination of sensitivity and specificity, which describes the inherent validity of a diagnostic test (Kumar and Indrayan, 2011).

### 2.7 Construction of ceRNA networks

The StarBase (version 3.0) database was used to search for the upstream target circRNA of miRNA (Li et al., 2014). Subsequently, a ceRNA network consisting of circRNA, miRNA and mRNA. Cytoscape was used to establish and visualize their relationships.

### 2.8 Immune infiltration correlation analysis

The CIBERSORT algorithm was used to calculate the infiltration of immune cells in COPD samples and normal samples based on the expression of GEO dataset (Chen et al., 2018). The visualization of immune cell richness was conducted using the R packages ggplot2 and ggpubr. Correlations between immune cells were analyzed using Pearson’s correlation coefficient, and immune cell correlation heatmaps were drawn using the corrplot package. The Lasso model was used to screen immune cells and chemokines closely related to the occurrence of COPD, and the screening criterion was lambda value were 0.025 and 0.027, respectively. Pearson’s correlation coefficient results between chemokines and immune cells were visualized using heat maps in R, utilizing the corrplot package.

### 2.9 Construction of COPD cell model

Different concentrations (1%, 5%, and 7.5%) of cigarette smoke extracts (CSE) were injected into a serum-free DMEM medium, with the pH adjusted to 7. A filter membrane (0.22 μm) was used to remove bacteria and particulate matter. The optical density (OD) value at 320 nm was measured for standardization of CSE. Seed logarithmically growing human airway epithelial cells (BEAS-2B) into five different concentrations of CSE DMEM medium. The absorbance values of each pore were measured using a Multiskan Spectrum Microplate Spectrophotometer (λ = 450 nm) to screen for the most suitable COPD cell model.

### 2.10 Real-time quantitative PCR assay

The expression of key RNAs in the ceRNA network was detected using real-time reverse transcription-PCR. RNA was extracted from the constructed COPD cell model using Trizol reagent (Accurate Biology, China), followed by reverse transcription. Real-time fluorescence quantitative PCR assays were performed using 2×NovoStart^®^ SYBR High-Sensitivity qPCR SuperMix (TIANGEN, China) as the fluorescence quantitative assay reagent, according to the instructions.

### 2.11 Statistical analyses

Data analysis and statistical tests were performed using R software (version 4.0.2, https://www.r-project.org/) and IBM SPSS Statistics. Pearson’s correlation coefficient was used for the correlation analysis. For normally distributed measurement data, a *t*-test for independent samples was used to determine whether the differences were significant. For non-normally distributed measurement data, the Mann-Whitney U test (i.e., Wilcoxon rank-sum test) is used to determine significant differences. Statistical significance is indicated by a *p*-value of less than 0.05.

## 3 Results

### 3.1 Identification of DEGs

**FIGURE 1:**
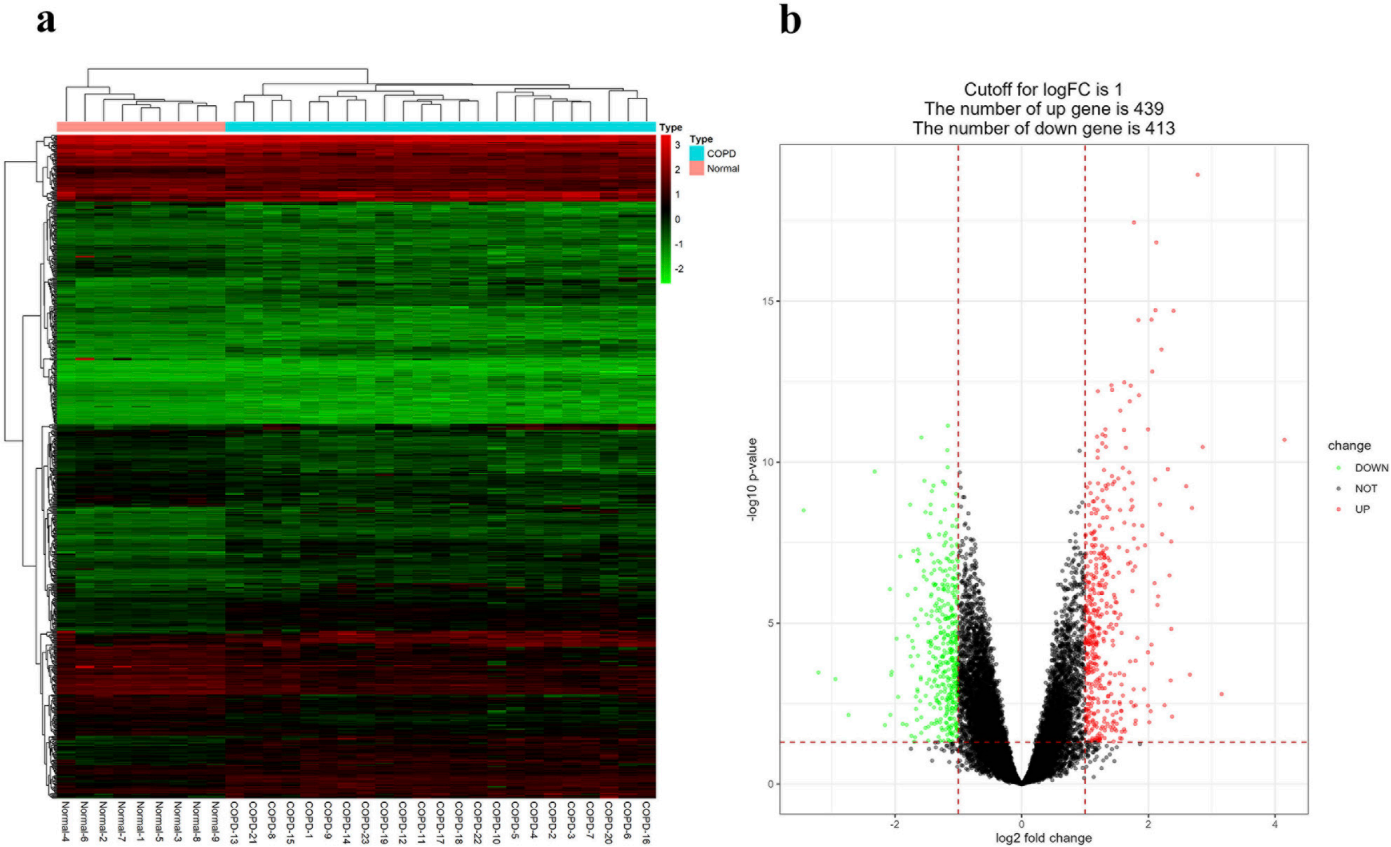
Identification of DEGs. **(A)** DEGs heatmap between COPD and normal samples. Red rectangles indicate high expression and green rectangles indicate low expression. **(B)** DEGs volcano plot between COPD and normal samples. The red graph shows upregulated genes, the black graph shows non-significant genes, and the green graph shows downregulated genes.

The GSE38974 dataset included 23 COPD samples and 9 normal samples t for the analysis and identification of DEGs. Compared to normal samples, a total of 852 DEGs were identified in COPD samples, including 439 upregulated DEGs and 413 downregulated DEGs. Then, these DEGs were then visualized using heatmap and volcano maps, as shown in Figures 1A, B.

### 3.2 Enrichment analysis of differentially expressed genes

**FIGURE 2:**
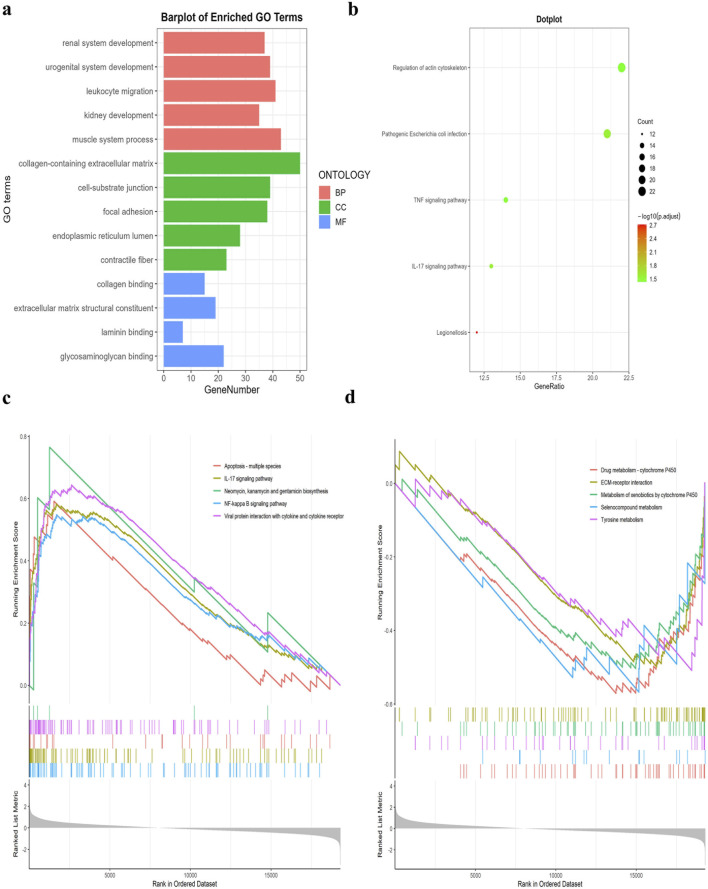
GO, KEGG pathway, and GSEA enrichment analyses of DEGs. **(A)** GO enrichment analyses of DEGs (BP, biological process; CC, cellular component; MF, molecular function). **(B)** KEGG pathway enrichment analyses of DEGs. All significant KEGG pathways. **(C)** GSEA enrichment analysis of upregulated DEGs. **(D)** GSEA enrichment analysis of downregulated DEGs.

GO analysis results revealed that the co-significant DEGs were mainly enriched in leukocyte migration, kidney development, cell-substrate junction, collagen binding, laminin binding, and glycosaminoglycan binding (Figure 2A). KEGG pathway enrichment analysis results showed that the co-significant DEGs were mainly enriched in the TNF signaling pathway, pathogenic *escherichia coli* infection, and the regulation of actin cytoskeleton (Figure 2B). The results of the GSEA enrichment analysis indicated that the pathways primarily enriched with upregulated genes were as follows: NF-kappa B signaling pathway, apoptosis, IL-17 signaling pathway, and interaction of viral proteins with cytokines and cytokine receptors. The downregulated genes exhibited significant enrichment in drug metabolism - cytochrome P450, ECM-receptor interaction, and tyrosine metabolism (Figures 2C, D).

### 3.3 PPI network construction, MCODE cluster modules and hub gene identification

**FIGURE 3:**
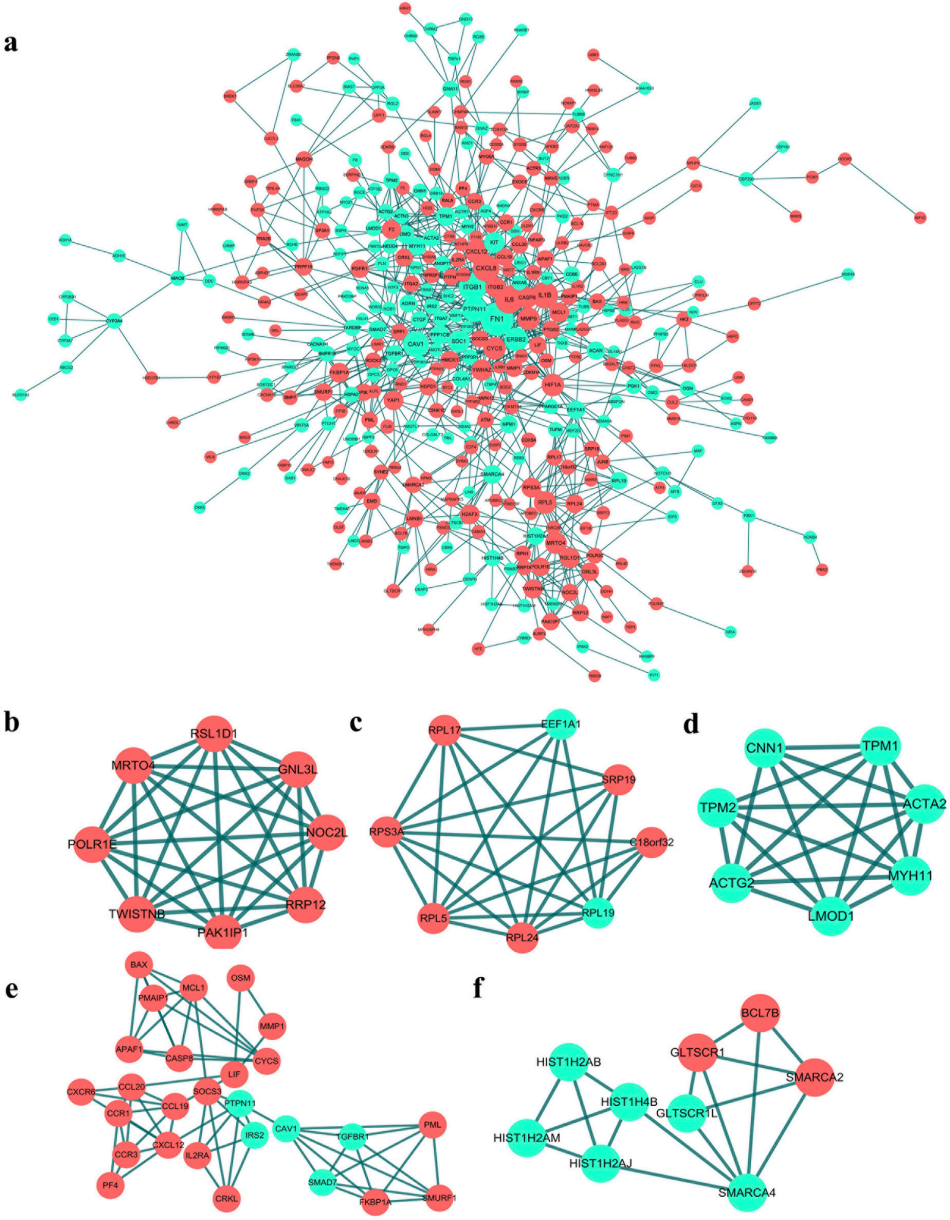
PPI network of DEGs and five key cluster modules. **(A)** PPI network of DEGs (Each node represents a protein, while each edge represents a protein-protein binding. Red circles represent upregulated genes and green circles represent downregulated genes). Five key cluster modules. Cluster 1 **(B)** had the highest cluster score (score:8.000, 8 nodes 28 edges), followed by cluster 2 **(C)** (score:6.857, 8 nodes 24 edges), cluster 3 **(D)** (score:6.667, 7 nodes 20 edges), cluster 4 **(E)** (score:4.923, 27 nodes 64 edges), and cluster 5 **(F)** (score:4.250, 9 nodes 17 edges).

We constructed the PPI network with DEGs associated with COPD by STRING, as shown in Figure 3A. A total of 399 DEGs associated with COPD and 866 PPI pairs were found. Next, we employed the MCODE plugin to perform clustering analysis and successfully identified five crucial modules within this network (Figures 3B–F). Cluster 1 (score: 8.000, 8 nodes 28 edges), Cluster 2 (score: 6.857, 8 nodes 24 edges), Cluster 3 (score: 6.667, 7 nodes 20 edges), Cluster 4 (score: 4.923, 27 nodes 64 edges), Cluster 5 (score: 4.250, 9 nodes 17 edges). Finally, we took the intersection of the results of five algorithms, Degree, MCC, MNC, DMNC, and cluster coefficient, performed in the cytohubba plugin. The seven hub genes, namely, PAK1IP1, NOC2L, RRP12, PMAIP1, CCL20, RPL17, and LIF, were identified.

### 3.4 Prediction of target miRNAs and construction of mRNA-miRNA co-expression networks

**FIGURE 4:**
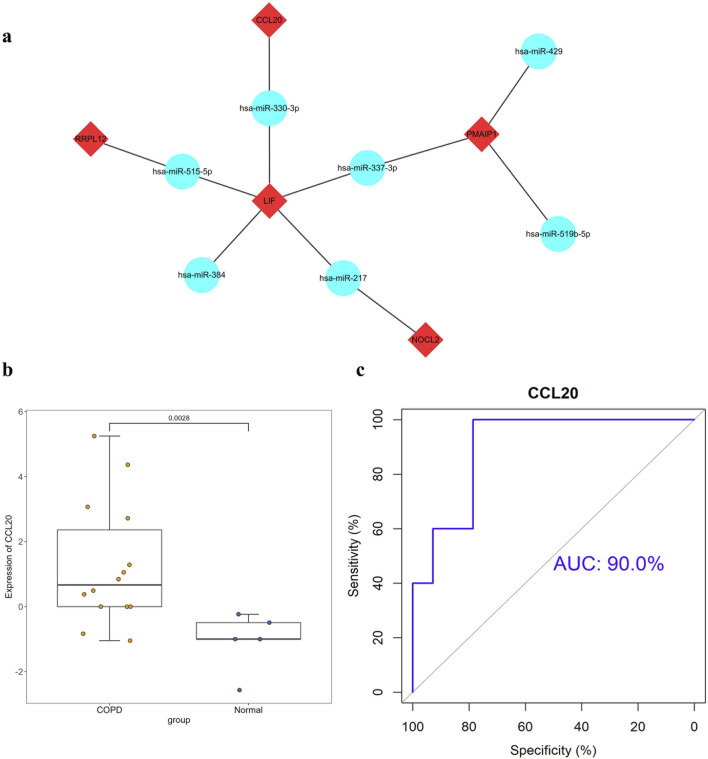
Construction of mRNA-miRNA co-expression networks and GEO database validation **(A)** mRNA-miRNA co-expression networks (a node represents an mRNA or miRNA, while an edge represents an interaction between mRNA and miRNA. Red diamond represents the hub gene and the blue circle represents the miRNAs). **(B)** Verification results of the hub gene CCL20(**: *p* < 0.01). **(C)** ROC curve of the hub gene CCL20[AUC:91.43%CI(0.7822-1)].

The hub genes were analyzed using the StarBase database to predict their corresponding target miRNAs, and the results of screening revealed seven shared target miRNAs corresponding to five hub genes (Figure 4A), which were as follows hsa-miR-337-3p, hsa-miR-217, hsa-miR-384, hsa-miR-515-5p, hsa-miR-519b-5p, hsa-miR-429.

### 3.5 GEO database validation and ROC curves for seven hub genes

The results of the validation of the GEO dataset showed that compared with normal samples, the mRNA expression level of CCL20 and RPL17 in COPD samples were significantly increased (*p* < 0.05), as shown in Figure 4B. ROC curve analysis showed that CCL20 had the highest diagnostic value in COPD samples (AUC: 0.900, 95%CI = 0.86-0.94), while RPL17 had a lower diagnostic value (AUC: 0.686, 95%CI = 0.62-0.75). Therefore, the combination of validation the GEO database and analysis of the result of ROC curve, we speculated that CCL20 could be served as a biomarker for the diagnosis of COPD (Figure 4C).

### 3.6 Target circRNA prediction and construction of ceRNA networks

**FIGURE 5:**
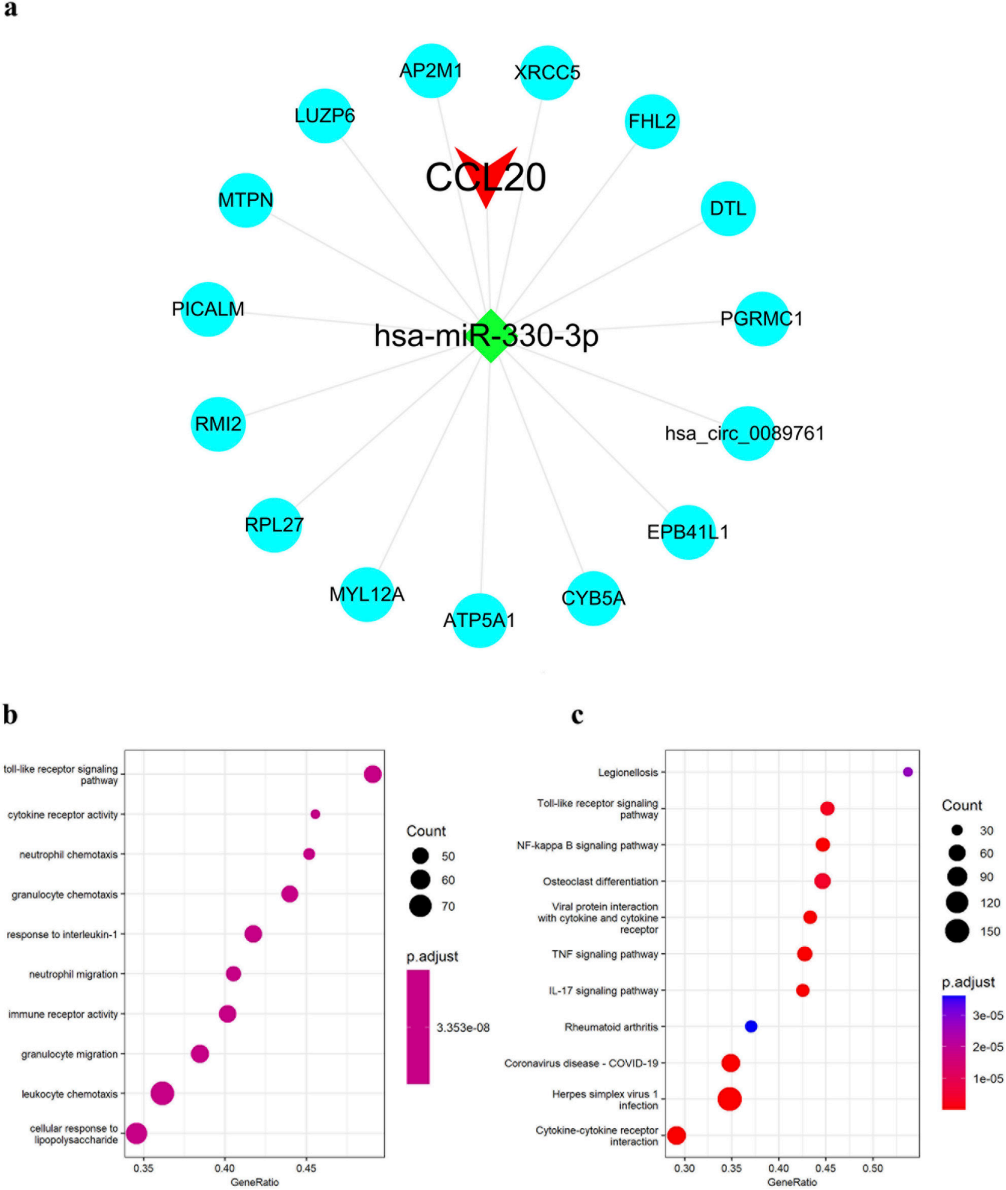
Construction of ceRNA networks of CCL20 and enrichment analysis of CCL20. **(A)** CCL20-associated ceRNA network. **(B)** GO enrichment analyses of CCL20. **(C)** KEGG pathway enrichment analyses of CCL20.

Based on the constructed miRNA-mRNA co-expression network, we used the StarBase database to retrieve CCL20 target hsa-miR-330-3p interacting circRNAs. Finally, 15 target circRNAs of the target hsa-miR-330-3p of CCL20 were obtained, and CCL20-associated ceRNA network was constructed, the network graph was visualized in Cytoscape (Figure 5A). Subsequently, we conducted a literature search based on the ceRNA hypothesis and found reports indicating that circDTL is associated with COPD (Lv et al., 2018; Shanshan et al., 2021). Hence, we suggested that the circDTL-hsa-miR-330-3p-CCL20 pathway could serve as a crucial regulatory pathway in the development of COPD. Additionally, the GO analysis revealed that CCL20 was primarily involved in cellular response to lipopolysaccharide, immune receptor activity, cytokine receptor activity, among others (Figure 5B). The KEGG analysis showed that CCL20 was primarily associated with signaling pathways closely related to cellular immune response, such as NF-kappa B signaling pathway and cytokine-cytokine receptor interaction (Figure 5C).

### 3.7 Correlation analysis of immune infiltration

**FIGURE 6:**
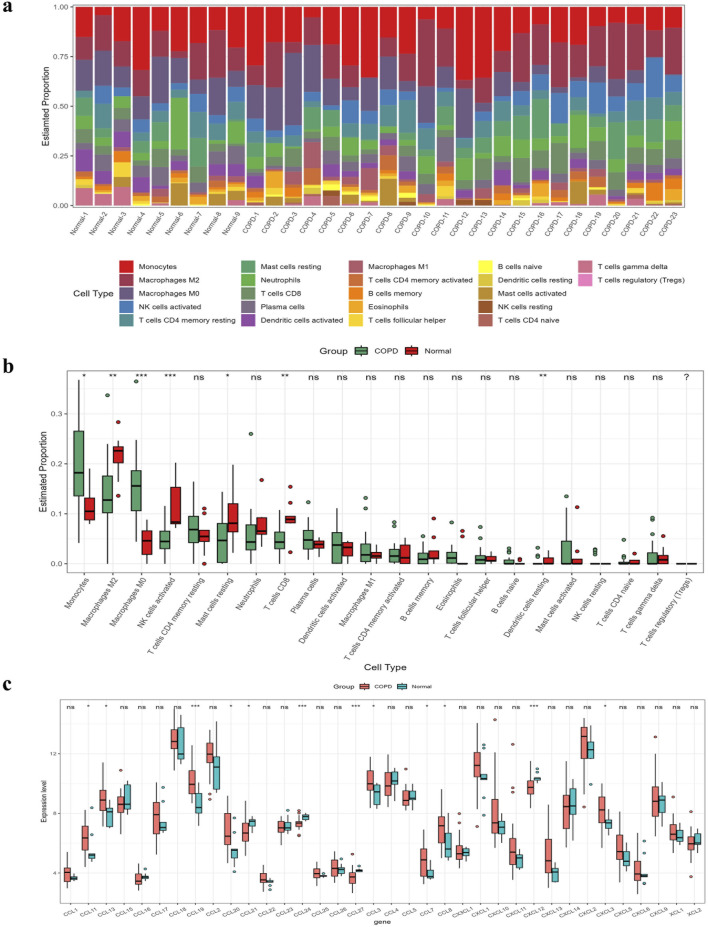
Calculation of abundance of immune infiltrating cells and evaluation of chemokines. **(A)** Abundance map of immune cells in different samples. **(B)** A comparison of the abundance of immune cells in COPD and normal samples. **(C)** Evaluation of 36 chemokines.

**FIGURE 7:**
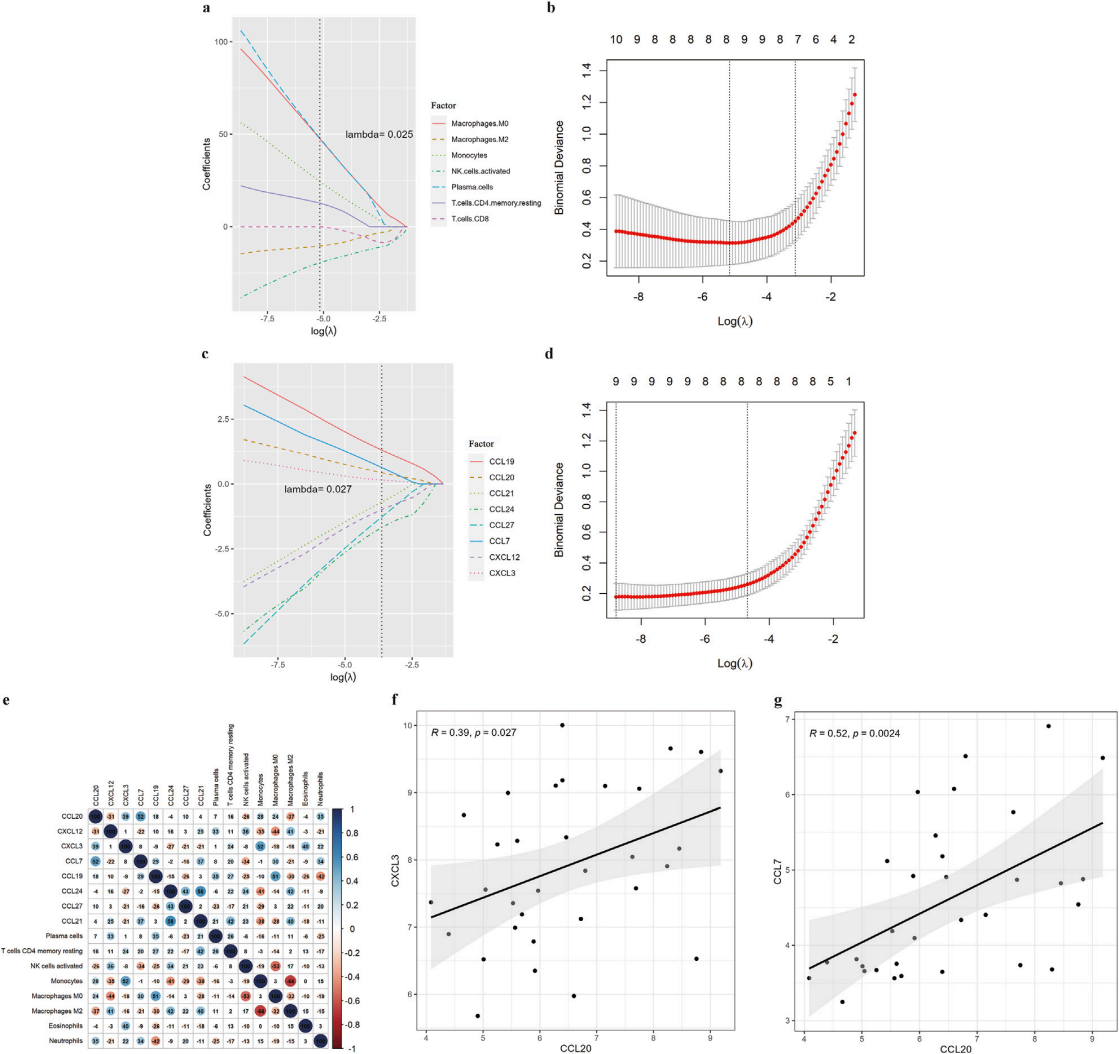
Correlation analysis of immune infiltration. **(A)** Lasso dimensionality reduction results for immune cells. **(B)** Lasso cross-validation results for immune cells. **(C)** Lasso dimension reduction results for significant chemokines. **(D)** Lasso cross-validation results for significant chemokines. **(E)** Correlation between chemokines and immune cells. **(F)** Correlation between hub gene CCL20 and chemokine CXCL3. **(G)** Correlation between hub gene CCL20 and chemokine CCL7.

As suggested by the enrichment analysis, the target genes might be involved in the immune response. Therefore, we first calculated the content of 22 immune cells (Figure 6A). The relative abundance of immune cells between COPD samples and normal samples was compared (Figure 6B). To identify chemokines associated with immune cell infiltration, we assessed the differential expression of 36 known human chemokines between COPD samples and normal samples (Figure 6C). Meanwhile, we used the Lasso model to analyze the immune infiltrating cells and chemokines (Figure 7). The results showed that eight types of immune infiltrating cells---Macrophages M0, Macrophages M1, Monocytes, Neutrophils, NK cells activated, Plasma cells, T cells CD4, T cells CD8 were could have a strong correlation with the progression of COPD (lambda value = 0.025). Additionally, eight chemokines (CCL19, CCL20, CCL21, CCL24, CCL27, CCL7, CXCL12, CXCL3) were found to be associated with COPD development (lambda value = 0.027, Figures 7A–D). Then, the correlation analysis results of chemokines and immune-infiltrating cells demonstrated that there was a noteworthy positive association between CCL20 and CCL7, and a negative correlation between CCL20 with macrophages M2 (Figure 7E). In addtion, the pearson correlation analysis showed that the expression level of CCL7 and CXCL3 were positively correlated with the expression level of CCL20 (Figures 7F, G).

### 3.8 RT-qPCR detection of ceRNA network expression

**FIGURE 8:**
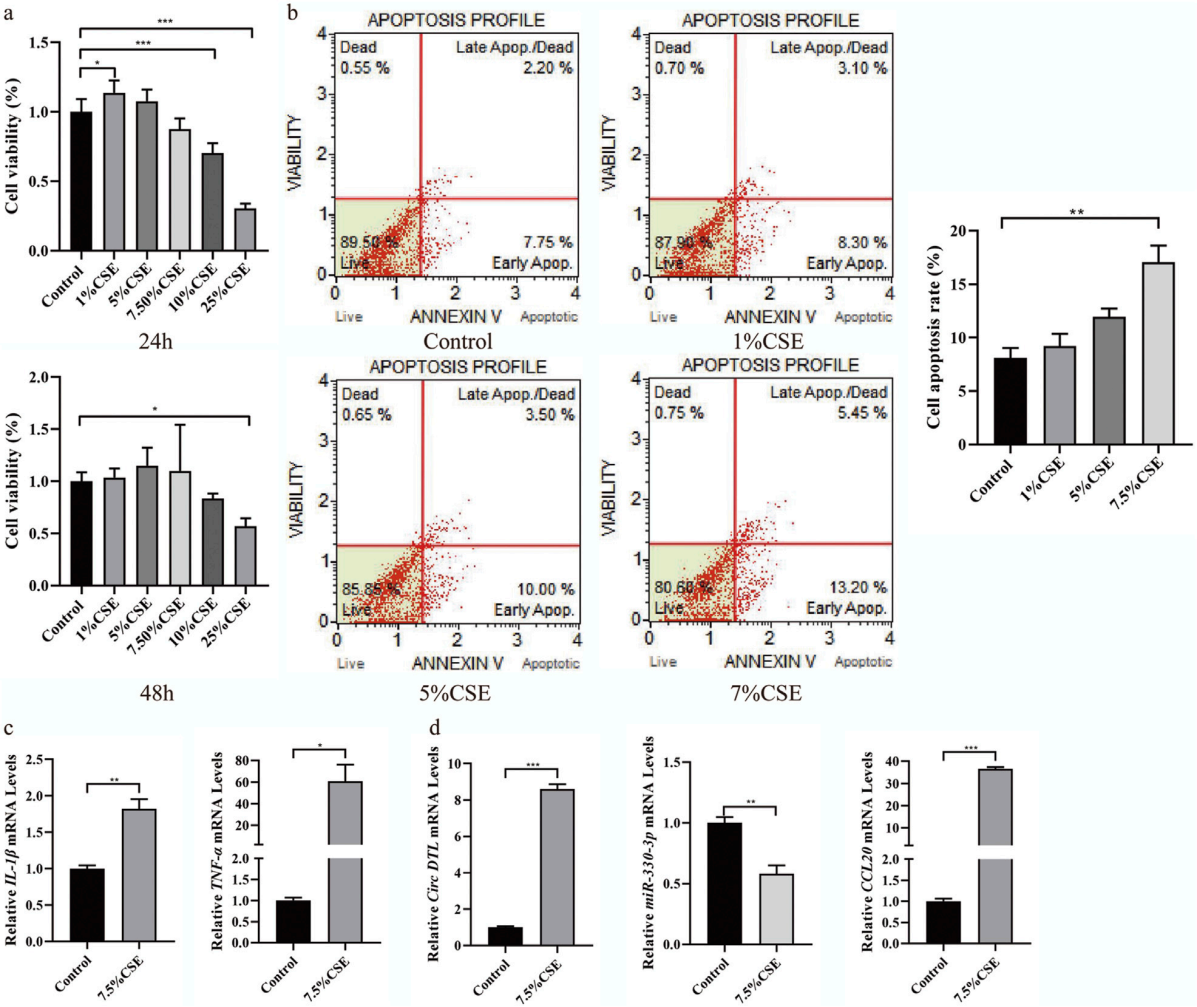
Construct the COPD cell model induced by CSE and validation of ceRNA network in the COPD cell model. **(A)** The cell proliferation was detected by CCK8 (24 and 48 h). **(B)** The cell apoptosis was detected by Muse. **(C)** The mRNA expression of inflammatory factors (Il-1β and TNF-α) was detected by RT-qPCR. **(D)** The expression level of circDTL, has-miR-330-3p and CCL20 were detected by RT-qPCR.

To investigate the impact of circDTL, hsa-miR-330-3p, and CCL20 on COPD cell function, we established a COPD cell model and treated BEAS-2B cells with 1%, 5%, and 7.5% cigarette extract (CSE) for 24 and 48 h. In Figure 8A, we observed that the optimal time of CSE was 24 h. And after treatment with 1%, 5%, and 7.5% CSE, the proportion of apoptosis was increased (9.95%, 11.40%, 13.50% and 18.65%, Figure 8B). Therefore, 7.5%CSE concentration was selected for the subsequent experiment.

Not only that, the increased expression of inflammatory factors (Il-1β and TNF-α) was observed in 7.5% CSE group in contrast with the control group, which mimics the phenomenon of airway inflammation in the COPD cell model (Figure 8C). RT-qPCR was used to measure the expression level of circDTL, hsa-miR-330-3p, and CCL20. The results showed that circDTL and CCL20 expression were increased while hsa-miR-330-3p was decreased in the COPD cell model (Figure 8D).

## 4 Discussion

The pathogenesis of COPD is complex and involves abnormalities in multiple cell types and molecular signaling pathways (Christenson et al., 2022). In recent years, the number of studies focusing on disease-associated RNAs has been rapidly increasing because the differential expression of specific genes is positively or negatively correlated with disease pathology. There is growing evidence that miRNAs, circRNAs, and mRNAs may exert a pivotal role in the pathogenesis of diverse diseases. Some of these molecules have been identified as potential biomarkers (Li et al., 2022). In this study, we identified the key gene CCL20 associated with COPD through bioinformatics analysis. Furthermore, we constructed the CCL20-associated with ceRNA network and explored its role in the pathogenesis of COPD, which has potential role in the early diagnosis, monitoring of disease progression and prognosis assessment of COPD.

circRNAs can competitively bind miRNAs, thus indirectly affecting the expression of target genes. In recent years, the relationship between circRNAs and respiratory diseases has become a research hotspot (Meng et al., 2022). Studies have shown that in COPD, circRNAs can influence the inflammatory response and apoptosis of airway epithelial cells by binding to hsa-miR-330-3p (Vos et al., 2016). The hsa-miR-330-3p is a miRNA encoded in the human genome, which plays an important role in regulating gene expression in cells. For instance, circ_0000003 is a decoy for miR-330-3p, and circ_0000003 participates in the progression of TSCC by sponging miR-330-3p (Qian et al., 2021). In this study, we identified the upregulation of circDTL and the downregulation of hsa-miR-330-3p. We verified this finding through the cellular experiments, which implicated circDTL in COPD progression by acting as a sponge for hsa-miR-330-3p.

Several studies have shown that hsa-miR-330-3p can be involved in a variety of inflammatory and immune response processes by targeting and regulating the expression of chemokines (Bai et al., 2023). Recently, researchers revealed that the ceRNA network may play a key role in the pathogenesis of COPD by regulating gene expression. A deeper understanding of these mechanisms could help us identify new therapeutic targets for COPD. For example, by targeting specific ceRNA molecules, it may be possible to modulate the inflammatory response and lung tissue damage associated with COPD (Firoozi et al., 2024). Therefore, in the present study, we identified CCL20, a potential target gene of hsa-miR-330-3p, through bioinformatics analysis, and constructed a ceRNA network (circDTL-hsa-miR-330-3p-CCL20). CCL20 is a chemokine that promotes the migration and aggregation of inflammatory cells, exacerbating inflammatory responses in the lungs (Boehme et al., 2016). In addition, the validation results of the GEO dataset and the analysis of curves suggested that CCL20 had potential to be a diagnostic biomarker for COPD. *In vitro* experiments, we observed that hsa-miR-330-3p expression was reduced in the COPD cell model, whereas in the expression of circDTL and CCL20 was increased. These results confirmed that circDTL interacted with hsa-miR-330-3p to regulate the expression of CCL20, which in turn triggered a variety of inflammatory and immune responses.

Furthermore, our analysis of immune cell infiltration and associated cytokines further underscored the centrality of CCL20 in COPD. Firstly, we identified eight immune-infiltrating cells closely associated with COPD (Macrophages M0, Macrophages M1, Monocytes, Neutrophils, NK cells activated, Plasma cells, T cells CD4 memory resting, and T cells CD8) and found that these cell types were correlated with eight chemokines (CCL19, CCL20, CCL21, CCL24, CCL27, CCL7, CXCL12, CXCL3). This result has shown that chemokines played a vital role in the regulation of immune cell infiltration and inflammation within lung tissue. Additionally, the correlation analysis results between CCL20 and eight key chemokines showed a noteworthy positive correlation between the expression level of CCL7 and CXCL3 with that of CCL20. This suggested that CCL20 may co-regulate the migration and infiltration of immune cells through these two chemokines. This interaction may have important implications for inflammatory response and immune regulation in COPD. Intervention in CCL20 may help control inflammation in COPD, and the treatment of COPD is expected to become a new strategy.

Although significant discoveries have been made, our research has certain limitations. Firstly, the datasets we selected have certain limitations. In the future, we will expand the sample size and conduct validation studies in different populations using RNA-Seq or single-cell RNA-Seq (scRNA-Seq) techniques to provide more comprehensive epidemiological data on COPD. Secondly, because this study mainly relied on samples of mixed tissues, we were not able to delve into the specific role of different cell types in the pathogenesis of COPD. Future studies should consider using higher throughput single-cell sequencing techniques to better analyze the effect of cell heterogeneity on COPD pathogenesis. Finaly, we completed cell experiments to study the expression of key genes in the ceRNA network associated with COPD by adding animal experiments.

## 5 Conclusion

In summary, this study successfully constructed a ceRNA regulatory network related to COPD (circDTL-hsa-miR-330-3p-CCL20) through biological information analysis and confirmed the interactions among key factors in the ceRNA network through cell experiments. The hub gene CCL20 expression was found to correlate with both prognosis and infiltration of immune cells in individuals diagnosed with COPD. The construction of the circDTL-hsa-miR-330-3p-CCL20 network in this research enhanced our comprehension of the molecular mechanisms involved in the onset and progression of COPD, while also providing important clues for the development of new therapeutic strategies and drug targets.

## Data Availability

The original contributions presented in the study are included in the article/supplementary material, further inquiries can be directed to the corresponding authors.
